# Preliminary results of official influenza and acute respiratory infection surveillance in two towns of Burkina Faso, 2013–2015

**DOI:** 10.1186/s12879-018-3241-3

**Published:** 2018-07-16

**Authors:** Tani Sagna, Abdoul Kader Ilboudo, Carine Wandaogo, Assana Cissé, Moussa Sana, Dieudonné Tialla, Armel Moumouni Sanou, David J. Muscatello, Zékiba Tarnagda

**Affiliations:** 1 Institut de Recherche en Sciences de la Santé (IRSS), Bobo-Dioulasso, Burkina Faso; 2 Ministère de la Santé, Ouagadougou, Burkina Faso; 3 School of Public Health and Community Medicine, UNSW Sydney, Sydney, Australia; 4 National Influenza Reference Laboratory, Unit of epidemic-prone diseases, emerging diseases and zoonoses, Department of Biomedical / Public health, Institute of research in health sciences, Bobo-Dioulasso, 01BP545 Burkina Faso

**Keywords:** Influenza, Burden, Influenza-like illness, Severe acute respiratory infections, Burkina Faso

## Abstract

**Background:**

In 2010, influenza, influenza-like illness (ILI) and acute respiratory infection (ARI) surveillance was established by the government of Burkina Faso. We provide preliminary descriptive results from this surveillance activity.

**Methods:**

The study period was 2013 through 2015. Two primary healthcare facilities in Bobo-Dioulasso district reported ILI in outpatients. Influenza virology, using reverse transcription-polymerase chain reaction (rRT-PCR), was available for a proportion of ILI patients. One hospital, in the capital Ouagadougou, reported ARI in both outpatients and inpatients (hospitalized). Inpatients admitted with ARI were considered severe ARI (SARI). We estimated the proportion of primary care outpatient visits that were ILI, and the proportion of those that were due to influenza, by age. We estimated the proportion of hospital outpatient visits that were ARI and the proportion of those that were SARI, by age.

**Results:**

Among combined outpatient visits in the Bobo-Dioulasso facilities, 19.6% were for ILI. One half (49.9%) of outpatient visits in infants and 30.9% in 1–4 year-olds were ILI. Among ILI outpatient visits 14.8% were due to influenza virus and, of these, 58.5% were type A and 41.5% type B. At the Ouagadougou hospital, 6.7% of outpatient visits were ARI, and 22.3% of those were SARI. The highest proportions of ARI were among infants (19.8%) and 1–4 year-olds (16.0%). The proportion of ARI that was SARI was highest among ≥15 year-olds (31.5%) followed by 1–4 year-olds (22.4%). Overall, 4.1% of SARI patients died.

**Conclusions:**

These preliminary data indicate the importance of respiratory infections among health care attendances in Burkina Faso, and influenza may be an important contributor to these.

## Background

According to the World Health Organization (WHO), seasonal influenza in humans is estimated to result annually in 3 to 5 million cases of severe illness and 290,000 to 650,000 deaths worldwide [1]. Among children, one in 10 hospitalizations for respiratory illness are estimated to be associated with influenza globally [2].

Burkina Faso lacks information on the epidemiology and burden of human influenza. In 2010, government sentinel surveillance for influenza commenced following the occurrence of pandemic influenza globally in 2009. Initial surveillance results confirmed the presence of circulating human influenza in the country. From 2010 to 2016, about 600 clinical swabs from all sites were analyzed per year by the Burkina Faso National Influenza Reference Laboratory (NIRL). During 2010–2012, the NIRL found that 6.6% of swabs collected from patients with influenza-like illness (ILI) were positive for influenza by virology [3].

We used selected influenza and respiratory infection surveillance sites that provided good data completeness to provide preliminary estimates of the proportional contribution of influenza-like illness, influenza and acute respiratory infections (ARI) to outpatient visits and hospitalizations for Burkina Faso.

## Methods

The study period was 2013 through 2015. The surveillance sites chosen for this study were selected due to completeness of reporting and our ability to access their health-care data. Health workers at the sites showed strong motivation in participating in this surveillance. The two cities targeted for this study are Ouagadougou and Bobo-Dioulasso. Bobo-Dioulasso is situated in the southwest of the country, some 350 km from the capital, Ouagadougou [4]. Public health care is organized into three levels; primary, secondary and tertiary, by the national Ministry of Health (MoH). In Ouagadougou there are five sanitary Districts (Bogodogo, Baskuy, Boulmigou, Nongr-Massom, Sig-Nonghin). Bobo-Dioulasso has two sanitary districts (Dafra and Dô). The two targeted ILI surveillance health centres provide primary care. Bolomakoté health centre is in Dafra District and Colsama health centre is in Dô District. We selected these centres due to their accessibility and the motivation of health workers in this surveillance. Secondary care services receive referrals from primary care health centres. The ARI surveillance site, Bogodogo District hospital, in Ouagadougou, provides secondary level care. Tertiary level care is provided by the national hospital center in Ouagadougou, which offers the highest reference level for specialized care [5].

Since 2010, active surveillance for ILI has been conducted at the two primary health care facilities. We regularly analyze oropharyngeal specimens for influenza screening from these sites. ILI was defined according to the World Health organization case definition: axillary temperature ≥ 38 °C, cough or sore throat and difficulty breathing within the last 10 days [6].

Real-time reverse transcription-polymerase chain reaction assay (rRT-PCR) was performed weekly only for specimens from the Bobo-Dioulasso sites. Respiratory specimens (oropharyngeal swabs) were collected from the first three patients meeting the ILI case definition during Monday to Thursday each week. Patients taking antivirals prior to presentation were excluded. We performed rRT-PCR using primers and probes according to the United States Centers for Disease Control and Prevention (US-CDC) protocol [7].

To estimate the proportion of ILI occurring in outpatient visits, we retrospectively analyzed combined surveillance data recorded during the period 2013 to 2015 from the two Bobo-Dioulasso health facilities. Age-specific proportions of total outpatient visits attributable to ILI were calculated, as were the annual and total proportion of ILI outpatient visits positive for influenza, and the proportion of influenza positive cases that were positive for influenza type A or B. This was done for persons of all ages and for the following age groups: < 1, 1–4, 5–14, ≥15 years.

Bogodogo health facility in Ouagadougou provided data on ARI outpatient visits, and hospitalized ARI only. We defined ARI requiring hospitalization as severe ARI (SARI). ARI was identified by medical record review. Respiratory sampling for acute respiratory infections only commenced at Bogodogo in 2014 and is not yet suitable for surveillance reporting. Medical records in Bogodogo are recorded in paper log books. This hospital has generally good and consistent data recording. Diagnoses are written in the logs and are not coded to a disease classification. We selected the following diagnosis groups as being ARI: rhinopharyngitis, (broncho)pneumonia, bronchitis, acute bronchiolitis, and other acute respiratory infections. At this site, we estimated the proportions of total outpatient visits that were ARI, the proportion of ARI that was SARI, and the hospital case fatality proportion among SARI.

## Results

Among 134,869 total outpatient visits in the two Bobo-Dioulasso primary care facilities during 2013 through 2015, 26,458 (19.6%) were for ILI. The ILI proportion decreased with increasing age, and 49.9% of children aged < 1 year presented with ILI (Table 1).

**Table 1:** Influenza-like illness (ILI) and total outpatient visits, two Bobo-Dioulasso facilities, by age, 2013–2015

Type of visit	Age group (years)				
	< 1 Number (%)	1–4 Number (%)	5–14 Number (%)	≥15 Number (%)	All ages Number (%)
ILI	6146 (49.9%)	8998 (30.9%)	3942 (15.4%)	7372 (10.9%)	26,458 (19.6%)
All outpatient visits	12,322 (100.0%)	29,163 (100.0%)	25,579 (100.0%)	67,805 (100.0%)	134,869 (100.0%)

Among the 26,458 ILI outpatient visits over the study period, rRT-PCR was performed for 1392, of which, 207 (14.9%) were positive for influenza. Among confirmed influenza ILI visits, 58.5% were type A and 41.5% were type B. The proportion of type B did not vary substantially across each year (Table 2).

**Table 2:** Influenza A and B among ILI outpatient visits with influenza virology, Bobo-Dioulasso facilties, Burkina Faso 2013–2015

Influenza type	Year			
	2013 Number (%)	2014 Number (%)	2015 Number (%)	2013–2015 Number (%)
Influenza A	61 (59.2%)	44 (56.4%)	16 (61.5%)	121 (58.5%)
Influenza B	42 (40.8%)	34 (43.6%)	10 (38.5%)	86 (41.5%)
Total influenza	103 (100.0%)	78 (100.0%)	26 (100.0%)	207 (100.0%)

**Fig. 1:**
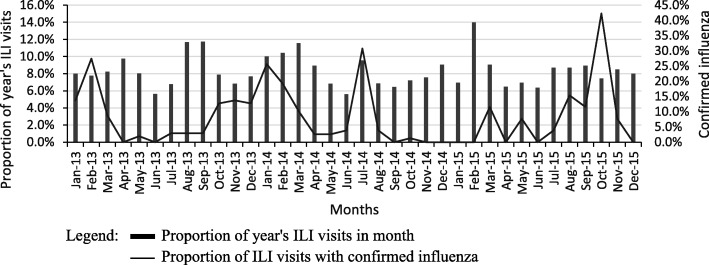
Outpatient visits with influenza-like illness (ILI) and influenza, by month, Bobo-Dioulasso facilities, 2013–2015

Figure 1 shows the trend in the proportion of each year’s ILI outpatient visits that occurred in each month from 2013 to 2015 for Bolomakoté and Colsama health centres combined. The proportion of virologically tested ILI visits that were positive for influenza are also shown. Peaks in ILI sometimes, but not always, corresponded to peaks in the proportion positive for influenza. Peaks in influenza occurred in the early months of each year, but other peaks also occurred as well as some periods of several months with sustained influenza activity.

During the study period, there were 115,655 outpatient visits to the Bogodogo facility, excluding medical records from July 2014 which could not be found. Among these, 7759 (6.7%) were ARI. The highest proportion of outpatient visits that were ARI was among infants (19.8%) followed by 1–4 year-olds (16.0%) (Table 3).

**Table 3:** (Severe) acute respiratory infections (ARI/SARI) among outpatient visits, by age, Bogodogo facility, 2013-2015^a^

Category	Age group (years)				
	< 1 Number (%)	1–4 Number (%)	5–14 Number (%)	≥15 Number (%)	All ages Number (%)
ARI	1769 (19.8%)	1968 (16.0%)	725 (5.8%)	3297 (4.0%)	7759 (6.7%)
SARI^b^	136 (7.7%)	441 (22.4%)	112 (15.4%)	1038 (31.5%)	1727 (22.3%)
Outpatients	8945 (100.0%)	12,262 (100.0%)	12,448 (100.0%)	82,000 (100.0%)	115,655 (100.0%)

^a^Excludes data not available for 1 month (July 2014)

^b^The denominator for SARI is ARI

Of the 7759 ARI visits, 1727 (22.3%) were hospitalized (SARI). The proportion of ARI that was SARI was highest among persons aged ≥15 years (31.5%) followed by 1–4 year olds (22.4%) (Table 3).

Among SARI patients, the hospital case fatality proportion was 4.1%. The case fatality proportion was highest among infants (6.6%) and lowest among 1–4 year-olds (1.4%) (Table 4).

**Table 4:** Severe acute respiratory infection deaths, by age, Bogodogo facility, 2013-2015^a^

Category	Age group (years)			
	< 1 Number (%)	1–4 Number (%)	≥5 Number (%)	All ages Number (%)
SARI deceased cases	9 (6.6%)	6 (1.4%)	55 (4.8%)	70 (4.1%)
All SARI	136 (100.0%)	441(100.0%)	1150(100.0%)	1727(100.0%)

^a^Excludes data not available for 1 month (July 2014)

## Discussion

During 2013 through 2015, influenza-like illness (ILI) was very common among patients presenting for outpatient visits in the two Bobo-Dioulasso facilities, with one in five experiencing ILI. ILI explained half of all visits in infants and almost one third of visits among children aged 1–4 years. Influenza accounted for around 15% of ILI outpatient visits. Among influenza ILI visits, influenza A accounted for around 59%. Overall, ARI accounted for around 7% of all outpatient presentations to Bogodogo district hospital but accounted for one in 5 presentations among infants and 16% among 1–4 year-olds. Just over one in five ARI presentations required hospitalization (SARI) and the proportion hospitalized was highest among patients aged 15 years and over – almost one third. Four per cent of SARI overall was fatal (in hospital), with a slightly higher proportion of fatalities among infants, over 1 in 20. The timing of ILI and ARI varied throughout the year, with unclear seasonality, although incidence appeared to be lowest during the middle of the year in the rainy season of Burkina Faso.

The proportion of ILI positive for influenza that we observed was similar to the proportion of 13% in Southwestern China during 2011–2015 using a similar case definition and CDC testing protocols [8]. Broadly similar proportions were found in Uganda, in 2016, with 13% [9]; in Niger during 2009 to 2013, with 12% [10], and Ghana during 2013 to 2015, with 18% [11]. Far higher proportions, between 28 and 37% were reported in Tunisia during 2013 to 2015 [12].

The seasonality of influenza during our 3 year study period was not clear, and data from additional years are needed to better identify seasonality. There is little influenza seasonality data for African regions. In Morocco, influenza peaked during the winter months of October through April [13]. In Nigeria and Niger, influenza peaked in February [10, 14]. In Ghana, cases increased in each of the two rainy seasons starting in May and August [15].

In our study, 6.7% of outpatient visits to Ouagadougou hospital were ARI. In Reunion, the rate was greater, about 11.9%, possibly due to the severe influenza season in that year (2016) [16]. We found a high proportion of hospital outpatient visits among children aged < 5 years were ARI and a high proportion of those required hospitalization. In 2015, Diene Sarr et al. in Senegal found that influenza was most frequent among children aged less than 24 months [17]. In China, among < 5 year-olds, children aged 6 months to 4 years experienced the highest rates of influenza-associated ILI [18]. Active surveillance in several developed and developing countries found picornaviruses (rhinovirus or enteroviruses), followed by influenza, were most commonly found in medically-attended and hospitalised children with ILI who were otherwise healthy [19]. In Ghana, the highest incidence rate of SARI was among children aged < 5 years [11]. A US study found that 10% of all children and 4% of the population aged < 65 years sought outpatient care for respiratory illness attributable to influenza annually [20]. Several other studies also revealed the involvement of ARI on outpatient visit among children [21–23]. These results highlight the strong involvement of respiratory infections in health-care attendance. While we were not able to estimate the age-specific contribution of influenza to either ILI or ARI, these international data suggest influenza is likely to be an important preventable cause of illness in young children in Burkina Faso. Immunization of children has already demonstrated it effectiveness against influenza infection [24–26]. Thus, free vaccination against influenza in children aged under 5 years may be an important preventive strategy in Burkina Faso.

We determined that case fatality proportion for ARI was highest among infants (6.6%) and lowest among 1–4 year-olds (1.4%). The average case fatality proportion was 4.1% among SARI cases. This is consistent with other studies demonstrating the importance of ARI as a cause of hospitalization and death [9, 18, 27].

Aside from the human disease burden of respiratory tract infection, there are economic [28–30], and social costs [31]. Thus, prevention of respiratory infections should be a priority.

There are substantial challenges to influenza surveillance in Burkina Faso. The quality of medical record keeping practices in health facilities is variable, and paper-based records are still the norm. Some medical records were unable to be located. Population catchment sizes for the health care facilities were unable to be estimated, and thus population rates were not able to be calculated.

## Conclusion

These preliminary influenza data indicate the strong involvement of respiratory infections among health care attendances in Burkina Faso, particularly among young children. Many require hospitalization and more than 1 in 20 infections in infants are fatal. Influenza appears to account for a substantial proportion of acute respiratory infections, although not the majority. We have demonstrated the feasibility of developing stronger influenza surveillance in Burkina Faso. These data will support the implementation of an influenza prevention and vaccine policy, particularly for young children.

## Abbreviations

ARI: Acute respiratory infection
ILI: Influenza-like illness
MoH: Ministry of Health
NIRL: National influenza reference laboratory
rRT-PCR: Real time reverse transcription-polymerase chain reaction
SARI: Severe acute respiratory infection
US-CDC: United States Centers for Disease Control and Prevention
WHO: World Health Organization
